# KNOWLEDGE, ATTITUDES, AND BELIEFS ABOUT HEARING HEALTH CARE OF OLDER BLACK WOMEN SEEKING HEARING HELP IN ENT SETTINGS

**DOI:** 10.1093/geroni/igad104.1471

**Published:** 2023-12-21

**Authors:** Julia Toman, Charity Lewis, Victora Sanchez, Michelle Arnold

**Affiliations:** University of South Florida, Tampa, Florida, United States; University of South Florida, Tampa, Florida, United States; University of South Florida, Tampa, Florida, United States; University of South Florida, Tampa, Florida, United States

## Abstract

There are documented barriers to access and use of healthcare services faced by older Black adults in the US. Black women are more likely to report negative past experiences with the healthcare system and as a result have inherent mistrust of providers. Black women are also less likely to receive referrals for specialty and allied health care, but it is unknown if this trend is apparent for hearing healthcare. The purpose of the current study was to investigate the experiences of older Black women seeking help for hearing loss in an otolaryngology (ENT) clinic setting. Our interdisciplinary team consisting of experts in gerontology and Black women’s health, public health, audiology, and otolaryngology conducted a mixed methods study which included (1) semi-structured interviews (n=15) and (2) online administration of a validated hearing beliefs questionnaire (n=86). Interview themes uncovered included experiences with racism in healthcare, the role of religion in help-seeking, and the importance of seeking treatment for hearing loss. Triangulation of findings revealed consistency between themes and questionnaire results, including little to no expressed stigma surrounding hearing aid use and little resistance to hearing healthcare or hearing loss treatment. Our results suggest that primary care and ENT should feel confident in referring older Black women who are seeking help for hearing loss to audiology for treatment options. We will also discuss other known facilitators and barriers to serving this population in an ENT setting.

